# CBFβ is a facultative Runx partner in the sea urchin embryo

**DOI:** 10.1186/1741-7007-4-4

**Published:** 2006-02-09

**Authors:** Anthony J Robertson, Carrie Dickey-Sims, Andrew Ransick, Dawn E Rupp, John J McCarthy, James A Coffman

**Affiliations:** 1 Stowers Institute for Medical Research, 1000 E. 50^th ^Street, Kansas City, MO 64110, USA; 2 Mount Desert Island Biological Laboratory, PO Box 35, Salisbury Cove, ME 04672, USA; 3 Division of Biology 156-29, California Institute of Technology, Pasadena, CA 91125, USA; 4 Department of Physiology, University of Kentucky College of Medicine, Lexington, Kentucky, 40506, USA

## Abstract

**Background:**

Runx proteins are developmentally important metazoan transcription factors that form a heterodimeric complex with the non-homologous protein Core Binding Factor β (CBFβ). CBFβ allosterically enhances Runx DNA binding but does not bind DNA itself. We report the initial characterization of SpCBFβ, the heterodimeric partner of SpRunt-1 from the sea urchin *Stronylocentrotus purpuratus*.

**Results:**

SpCBFβ is remarkably similar to its mammalian homologues, and like them it enhances the DNA binding of the Runt domain. SpCBFβ is entirely of zygotic provenance and its expression is similar that of SpRunt-1, accumulating globally at late blastula stage then later localizing to endoderm and oral ectoderm. Unlike SpRunt-1, however, SpCBFβ is enriched in the endodermal mid- and hindgut of the pluteus larva, and is not highly expressed in the foregut and ciliated band. We showed previously that morpholino antisense-mediated knockdown of SpRunt-1 leads to differentiation defects, as well as to extensive post-blastula stage apoptosis caused by under-expression of the Runx target gene *SpPKC1*. In contrast, we show here that knockdown of SpCBFβ does not negatively impact cell survival or *SpPKC1 *expression, although it does lead to differentiation defects similar to those associated with SpRunt-1 deficiency. Moreover, SpRunt-1 containing a single amino acid substitution that abolishes its ability to interact with SpCBFβ retains the ability to rescue cell survival in SpRunt-1 morphant embryos. Chromatin immunoprecipitation shows that while the *CyIIIa *promoter engages both proteins, the *SpPKC1 *promoter only engages SpRunt-1.

**Conclusion:**

SpCBFβ is a facultative Runx partner that appears to be required specifically for cell differentiation.

## Background

Runx proteins are transcription factors that function critically in gene regulatory networks that control cell proliferation and differentiation during animal development [1]. The Runx family is defined by a highly conserved 128 amino acid sequence known as the Runt domain, which binds specifically to the DNA sequence TG^T^/_C_GGT [2,3]. The sea urchin Runx transcription factor SpRunt-1 was discovered biochemically as a protein that binds this sequence in the *cis*-regulatory domain of the aboral ectoderm differentiation gene *CyIIIa*, an interaction that contributes to transcriptional activation of *CyIIIa *[4,5]. *SpRunt-1 *transcripts are globally expressed in the early embryo, and then become localized predominantly to regions of continued growth and cell proliferation within larval oral ectoderm and endomesoderm [6]. Morpholino antisense-mediated knockdown of SpRunt-1 has shown that it is required throughout the embryo for cell differentiation [7] and survival [8]. The latter function is mediated by a positively-acting interaction between SpRunt-1 and its target gene *SpPKC1*, which encodes a conventional protein kinase C [8].

Runx proteins heterodimerize with another protein commonly referred to as core binding factor beta (CBFβ), a non-DNA binding protein that allosterically enhances the DNA binding of the Runt domain [9]. Mammalian CBFβ also increases the half-life of Runx1 by protecting it from ubiquitin-mediated proteolysis [10]. Since Runx1 contributes to cell cycle control and its protein levels are cell cycle regulated [11], this suggests that CBFβ may be a regulatory subunit that modulates Runx activity during the developmental transition from proliferation to terminal differentiation.

Knockout of *CBFb *in mouse leads to embryonic lethality caused by a failure of hematopoiesis [12,13], a phenotype very similar to that caused by knockout of *Runx1*. As with *RUNX1*, chromosomal translocations involving *CBFb *are commonly associated with human leukemia [9]. Like *Runx2*, *CBFb *has also been shown to be important for osteogenesis, although its loss-of-function phenotype is not identical to that caused by *Runx2 *knockout since some osteoblast differentiation does occur [14,15]. *Drosophila *has two CBFβ genes, *brother *and *big brother*, the products of which contribute redundantly to the functionality of the Runx proteins Runt and Lozenge [9]. These studies all indicate that CBFβ is important for Runx function; however, to date, no studies have definitively addressed the question of whether the Runx-CBFβ interaction is constitutive (obligate or context-independent) or facultative (regulatory or context dependent). The latter possibility is suggested by the facts that (a) the interaction is inhibited by sequences within mammalian Runx2 [16] and the C-terminal domains of some Runx1 isoforms [17], and (b) Runx proteins can bind DNA as a monomer, albeit less effectively than as a heterodimer [18]. Mouse CBFβ, which is ubiquitously expressed, has been shown to interact specifically with another protein termed Crl-1, which is expressed in subsets of neuronal cells [19]. Finally, avian *CBFb *displays a complex pattern of expression in early development that is not identical to the pattern of *Runx1 *expression, with some regions of the embryo expressing only one or the other gene [20]. Taken together, these observations invite speculation that Runx proteins might interact with only a subset of target genes (or with some target genes only part of the time) as a heterodimer with CBFβ, and that the choice between these alternative states may in part be determined by the specific Runx isoform as well as protein-protein interactions specified by the context of each particular tissue, cell type, and *cis*-regulatory system.

Here we describe the cloning and initial characterization of SpCBFβ, the heterodimeric Runx partner from the sea urchin *Strongylocentrotus purpuratus*. We show that SpCBFβ is expressed in a pattern that is similar but not identical to that of SpRunt-1. Moreover, while SpCBFβ participates in the Runx-dependent activation of several genes including *CyIIIa*, it is not required for or involved in Runx-dependent activation of *SpPKC1*, which we showed previously to be critical for cell survival in the embryo [8]. Therefore, heterodimerization of SpRunt-1 with SpCBFβ occurs facultatively in the sea urchin, and is a context-dependent aspect of Runx-mediated transcriptional control.

## Results and Discussion

### SpCBFβ expression during embryogenesis

**Figure 1:**
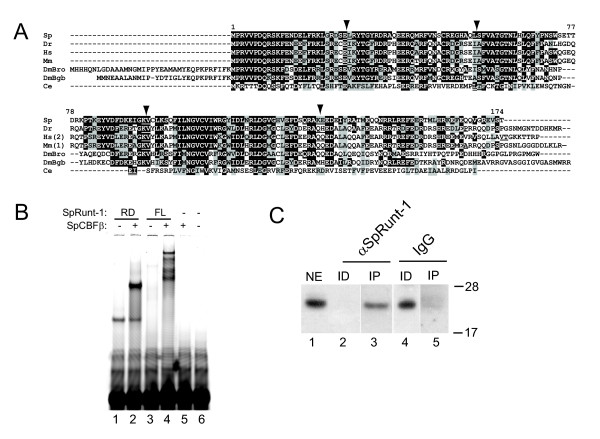
**SpCBFβ structure and heterodimerization with SpRunt-1**. (A) Alignment of CBFβ sequences from *S. purpuratus* (Sp) zebrafish (Dr), *Homo sapiens* (Hs, variant 2 of the two mammalian splice variants), mouse (Mm, variant 1 of the two mammalian splice variants), *Drosophila* (two genes, DmBro and DmBgb), and *C. elegans*.  Amino acid residues that are identical between SpCBFβ and at least one other homologue are highlighted in black, and conserved substitutions are highlighted in grey.  Amino acid pairs of which the respective codons are separated by introns in each gene are underlined; the four introns conserved between urchin and vertebrates are indicated by arrowheads.  (B) EMSA analysis using recombinant Runt domain (RD) or full-length SpRunt-1 (FL) either alone or in the presence of recombinant SpCBFβ.  Note that the full-length SpRunt-1 does not bind DNA very effectively by itself, suggesting the presence of domains that inhibit DNA binding within the full length protein.  SpCBFβ complexes with both the Runt domain and full-length SpRunt-1, as indicated by a “supershift”, but does not bind DNA by itself.  (C) Immunoblot of SpCBFβ from nuclear extract (NE), and nuclear extract immundepleted (ID) and immunoprecipitated (IP) with anti-SpRunt-1 or nonspecific IgG.  Molecular weight markers (in kilodaltons) are shown on the right.

Sequences encoding the sea urchin homologue of CBFβ were amplified from *S. purpuratus *blastula stage cDNA by polymerase chain reaction (PCR) using degenerate primers corresponding to highly conserved regions of the protein. The resulting amplicon was cloned and sequenced, and the sequence was used to obtain the full-length SpCBFβ mRNA by 5' and 3' RACE. The SpCBFβ mRNA encodes a protein with a predicted molecular weight of ~21 kDa. As shown in Fig. 1A, the deduced amino acid sequence of SpCBFβ is highly similar to that of its homologues from other phyla, and as would be expected, is more similar to the vertebrate representatives than to those from the non-deuterostome invertebrates. *SpCBFbeta *gene sequences were located in the *S. purpuratus *genome database [21], and the exon-intron structure of the gene was deduced from alignment with the cDNA. *SpCBFbeta *appears to be the only homologue in the *S. purpuratus *genome, and contains four introns, the positions of which are conserved in vertebrates (Fig. 1A). In contrast, the *Drosophila *genes *bro *and *bgb *have no introns, while the *C. elegans *homologue is missing introns 1 and 4 (with its second intron displaced upstream in the coding sequence with respect to intron 3 of the deuterostome homologues; Fig. 1A).

Electrophoretic mobility shift analysis (EMSA) was used to test whether SpCBFβ enhances the DNA binding activity of SpRunt-1. Recombinant SpRunt-1 protein was reacted with a fluorescently labeled oligonucleotide probe containing the SpRunt-1 binding site from *CyIIIa *[5], either with or without pre-incubation with recombinant SpCBFβ. DNA binding of full-length SpRunt-1, or a fragment containing only the Runt domain thereof, is substantially enhanced in the presence of SpCBFβ, which forms a complex with the SpRunt-1 protein (as indicated by a "supershift"; Fig. 1B, lanes 2 and 4), whereas SpCBFβ alone does not bind DNA (Fig. 1B, lane 5). It is likely that native SpRunt-1 forms a heterodimeric complex with SpCBFβ in the embryo, and consistent with this, affinity purified SpRunt-1 protein from blastula stage nuclear extracts was initially identified as a heterodimer containing a 21 kDa subunit [4]. To confirm this, an antibody generated against recombinant SpCBFβ was used to probe an immunoblot of whole nuclear extract, nuclear extract immunodepleted with an anti-SpRunt-1 antibody, and the resulting immunoprecipitate. A 21 kDa SpCBFβ-immunoreactive band present in blastula stage nuclear extract (Fig. 1C, lane 1) is specifically immunodepleted from the extract by the SpRunt-1 antibody (Fig. 1C, lane 2), and pulled down in the SpRunt-1 immuoprecipitate (Fig. 1C, lane 3), whereas the band is not similarly precipitated by non-specific IgG (Fig. 1C, lanes 4 and 5). These data suggest that SpCBFβ exists in a complex with SpRunt-1 *in vivo*.

**Figure 2:**
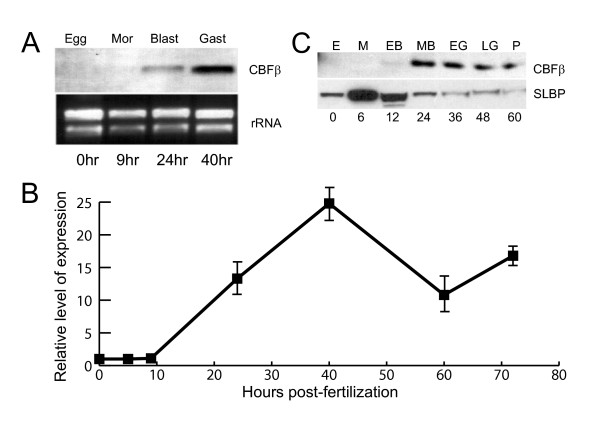
**Temporal expression of SpCBFβ**. (A) Northern blot of total RNA from egg, morula, blastula, and gastrula stage embryos probed with SpCBFβ. The ethidium bromide stained rRNA bands from the same gel are shown as a loading control. (B) Temporal expression of SpCBFβ as measured by RT-PCR. (C) Immunoblot of equivalent amounts of total protein from egg (E), morula (M), early blastula (EB), mesenchyme blastula (MB), early gastrula (EG), late gastrula (LG) and pluteus stage (P), probed with antibodies to either SpCBFβ or SLBP (a positive control for the presence of intact protein in the egg and early embryo extracts).

RNA blot analysis shows that SpCBFβ is represented by a single species of transcript that is virtually absent in the egg and early embryo, and which accumulates dramatically during blastula and gastrula stages (Fig. 2A). The temporal expression of SpCBFβ was further examined by quantitative reverse transcription-coupled PCR (qRT-PCR). SpCBFβ transcripts are present at low levels during cleavage, accumulate approximately 25-fold between 9 and 40 hours post-fertilization (hpf), and thereafter decline in abundance (Fig. 2B). This pattern parallels that of SpRunt-1 [5], although possibly with a slight temporal lag. Immunoblot analysis (Fig. 2C) reveals that there is no maternal SpCBFβ protein, and that zygotic accumulation of SpCBFβ protein is similar to that of its mRNA, although unlike the mRNA the protein levels do not increase significantly between 24 and 40 h, suggesting that there may be translational or post-translational regulation during this interval.

Quantitative RT-PCR was also used to measure the abundance of SpCBFβ transcripts. Toward this end a standard curve was constructed from in vitro synthesized SpCBFβ mRNA (see Methods). We used the same method to measure the abundance of SpRunt-1 transcripts, which were previously measured by an RNAase protection method [5]. The measurements of SpRunt-1 were in agreement with our previous measurements, indicating 7,000–8,000 transcripts per blastula or gastrula stage embryo. In the mid-gastrula stage embryo, when SpCBFβ is maximally expressed, it is present at slightly higher levels than is SpRunt-1, determined by qRT-PCR to be 12,000 transcripts per embryo. Thereafter, SpCBFβ transcripts decline in abundance to levels approximating those of SpRunt-1.

**Figure 3:**
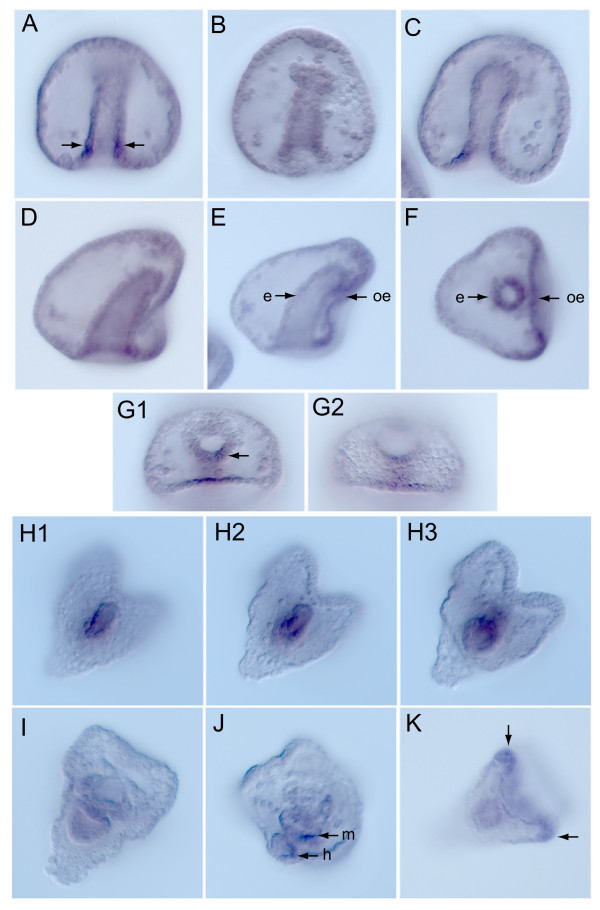
**Spatial expression of SpCBFβ**. Whole mount in situ hybridization of (A-G) Two day old gastrula stage embryos showing global expression, with enrichment in endoderm (e) and parts of the oral ectoderm (oe), and (H-K) four day old pluteus showing expression localized to midgut (m) and hindgut (h) endoderm, and some expression in the ciliated band of the anal arm buds (K, arrows).

The spatial pattern of SpCBFβ expression was examined by whole mount in situ hybridization (WMISH) (Fig. 3). As with its temporal expression, SpCBFβ is expressed initially in a spatial pattern that is similar to that of SpRunt-1: at mesenchyme blastula stage, transcripts are globally distributed (not shown), and by late gastrula stage, they start to become enriched in the oral ectoderm and endomesderm (Fig. 3A–G; arrows in 3E, F). In the 2-day old gastrula endoderm expression is highest in a ring of cells just inside the blastopore (Fig. 3A–C; arrows in 3A). Expression is also enhanced in the oral ectoderm (Fig. 3D–F) and on the oral side of the hindgut region (Fig. 3G, arrow). In the mature (4-day) pluteus, SpCBFβ transcripts are expressed most prominently in the differentiating midgut and hindgut of the endoderm (Fig. 3H–J; arrows in 3J), whereas in the ciliated band, expression is confined to the tips of the anal arm buds (Fig. 3K, arrows). This is somewhat different from the larval expression pattern of SpRunt-1, which is also expressed in the endoderm but most prominently in the larval foregut region, as well as throughout the ciliated band [6].

### SpCBFβ is dispensable for the pro-survival function of SpRunt-1

**Figure 4:**
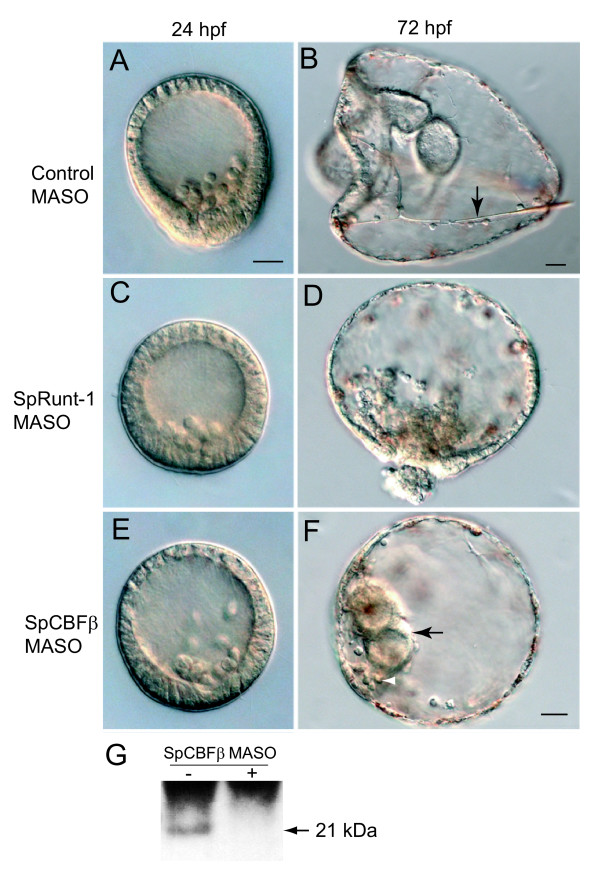
**Comparison of SpCBFβ and SpRunt-1 morphant phenotypes**. DIC images of (A) 24 h and (B) 72 h control MASO injected embryos; arrow in (B) points to skeletal rod (spicule); (C) 24 h and (D) 72 h SpRunt-1 MASO injected embryos; and (E) 24 h and (F) 72 h SpCBFβ MASO injected embryos; black arrow in (F) points to tripartite gut, and white arrowhead points to a "ventrolateral" cluster of skeletogenic mesenchyme cells. Scale bars = 20 μm; embryos in (A), (C), (D) and (E) are to the same scale. (G) Immunoblot of total blastula-stage embryo extract showing that the SpCBFβ MASO is effective in depleting endogenous SpCBFβ (arrow).

A morpholino antisense oligonucleotide (MASO) was designed to target the translational start site in the SpCBFβ mRNA. Introduction of this MASO into embryos produces a distinctive phenotype that is milder than that obtained with SpRunt-1 MASOs (Fig. 4). While SpCBFβ morphants display morphological defects, most notably a failure of skeletogenesis and a poorly differentiated ectoderm (Fig. 4F and see gene expression analysis below), unlike SpRunt-1 morphants, they gastrulate (Fig. 4F, arrow). The SpCBFβ morphants also have a secondary axis and appear to form bilateral clusters of skeletogenic mesenchyme cells (Fig. 4F, arrowhead), which normally occurs in response to signals from the oral ectoderm [22], suggesting that the ectodermal and skeletogenic differentiation defects are not caused by a failure of regional specification, but rather of subsequent cell differentiation. That the SpCBFβ MASO effectively depletes embryos of SpCBFβ is shown by immunoblot analysis of endogenous protein (Fig. 4G).

**Figure 5:**
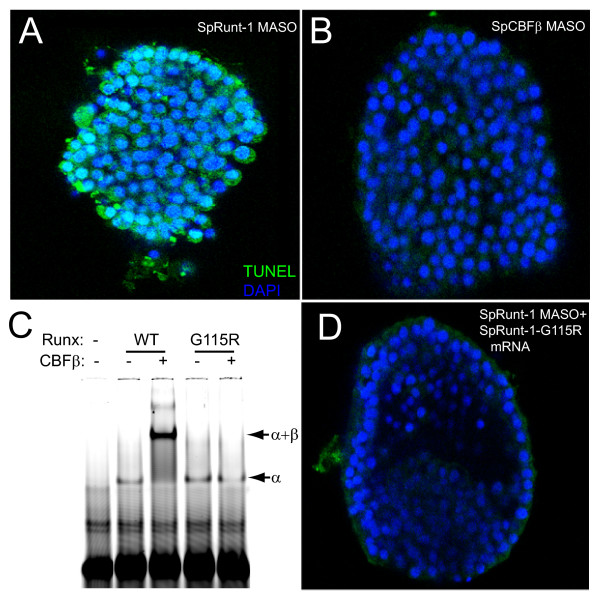
**SpCBFβ does not contribute to the anti-apoptotic function of SpRunt-1**. (A) Gastrula stage SpRunt-1 morphant fluorescently imaged with TUNEL (green, indicating apoptosis) and DAPI (blue, indicating DNA). (B) Gastrula stage SpCBFβ morphant fluorescently imaged with TUNEL and DAPI as in (A). (C) EMSA of recombinant wild-type and mutant (G115R) Runt domain (α) from SpRunt-1, in the presence or absence of recombinant SpCBFβ. Note that while the G115R mutant Runt domain binds DNA, it does not heterodimerize with SpCBFβ. (D) Gastrula stage SpRunt-1 morphant that has been co-injected with full-length mRNA encoding SpRunt-1-G115R, fluorescently imaged with TUNEL and DAPI as in (A). Note that while cell survival is rescued, as indicated by a lack of TUNEL signal, other aspects of development such as skeletogenesis are not.

The gastrulation defective phenotype associated with SpRunt-1 deficiency is largely a secondary effect of extensive apoptosis that occurs throughout the post-blastula stage embryo [8]. Since SpCBFβ morphants gastrulate, we hypothesized that they do not undergo the extensive apoptosis characteristic of SpRunt-1 morphants. This is indeed the case: unlike embryos injected with the SpRunt-1 MASO (Fig. 5A), those injected with the SpCBFβ MASO display negligible TUNEL signal (Fig. 5B). This suggests that SpCBFβ is dispensable for SpRunt-1 function in promoting cell survival.

SpRunt-1 derived from exogenous mRNA rescues cell survival in SpRunt-1 morphants [8]. If SpCBFβ is dispensable for the survival function of SpRunt-1, then a SpRunt-1 protein that has been mutated so that it cannot interact with SpCBFβ should retain the ability to rescue cell survival in SpRunt-1 morphants. To test this, we constructed a SpRunt-1 mutant protein wherein an arginine is substituted for a glycine residue that is essential for the interaction between the Runt domain and CBFβ (SpRunt-1-G115R). Electrophoretic mobility shift analysis verifies that the Runt domain from SpRunt-1-G115R retains the ability to bind DNA as a monomer, but does not heterodimerize with SpCBFβ (Fig. 5C). Importantly, SpRunt-1-G115R rescues cell survival in SpRunt-1 morphants (Fig. 5D), indicating that heterodimerization with SpCBFβ is not required for the anti-apoptotic function of SpRunt-1.

### *SpPKC1 *is a SpCBFβ-independent Runx target

**Figure 6:**
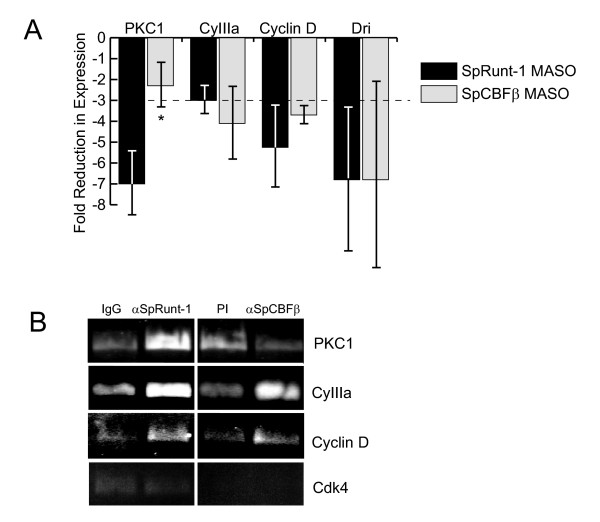
**SpCBFβ is not involved in Runx-mediated regulation of *SpPKC1***. (A) Relative expression levels of *SpPKC1*, *CyIIIa*, *SpCyclinD *and *SpDri *as determined by RT-PCR analysis of RNA isolated from gastrula stage SpRunt-1-knockdown and SpCBFβ-knockdown embryos. Each bar represents the average +/- standard deviation in the level of expression with respect to controls from two or more microinjection experiments. The dashed line indicates a 3-fold reduction of expression, which is taken here to be the minimum level of reduction considered to be significant. Note that whereas the expression of *CyIIIa*, *SpCyclinD *and *SpDri *are similarly affected by depletion of either SpRunt-1 or SpCBFβ, expression of SpPKC1 is much more strongly affected by SpRunt-1 depletion than by SpCBFβ depletion (asterisk). (B) ChIP of *CyIIIa*, *SpPKC1*, *SpCyclinD *and *SpCdk4 *using anti-SpRunt-1 and anti-SpCBFβ antibodies and chromatin prepared from late gastrula stage (48 h) embryos. A product of the expected size was obtained from the input DNA with each primer set, confirming the efficacy of the primers and PCR conditions (not shown). *SpCdk4*, which was not recovered by ChIP with either antibody, was chosen as a specificity control because its expression levels are not affected by knockdown of either SpRunt-1 or SpCBFβ.

The apoptotic phenotype in SpRunt-1 deficient embryos is specifically caused by a deficit in the expression of *SpPKC1*, a direct regulatory target of SpRunt-1 that encodes a conventional protein kinase C [8]. The fact that SpCBFβ-deficient embryos do not display an apoptotic phenotype suggests that loss of SpCBFβ function does not adversely affect the expression of *SpPKC1*. To test this we used reverse transcription-coupled PCR to measure the relative levels of *SpPKC1 *transcripts in either SpRunt-1 morphants or SpCBFβ morphants with respect to controls. Whereas *SpPKC1 *is ~7-fold under-expressed in SpRunt-1-deficient gastrula stage embryos, its expression at that stage is not significantly affected by knockdown of SpCBFβ (Fig. 6A). In contrast, expression of the aboral ectoderm differentiation marker *CyIIIa *is diminished to an equivalent extent by knockdown of either SpRunt-1 or SpCBFβ, as are expression of two other putative SpRunt-1 targets [7], *SpCyclinD *and *SpDri *(the latter encodes a transcription factor required both for oral ectoderm differentiation and skeletogenesis [23]) (Fig. 6A). These results indicate that at gastrula stage, SpRunt-1-mediated activation of *CyIIIa*, *SpCyclinD *and *SpDri *requires heterodimerization with SpCBFβ, whereas SpRunt-1-mediated activation of *SpPKC1 *does not. Interestingly, *CyIIIa *expression is not as strongly affected by SpRunt-1 depletion as is *SpPKC1 *expression; this could relate to the fact that the splice-blocking MASO does not completely deplete SpRunt-1 protein [7], some of which is provided maternally [24]. These data suggest that *SpPKC1 *activity is particularly sensitive to SpRunt-1 protein levels, which might occur if the architectural context of the *SpPKC1 cis*-regulatory system confers selectivity for zygotically-expressed SpRunt-1 and/or SpRunt-1 isoforms that cannot heterodimerize with SpCBFβ.

While these results indicate that SpCBFβ is not required for the transcriptional activation of *SpPKC1*, they do not rule out the possibility that SpCBFβ nonetheless heterodimerizes with SpRunt-1 on its target sequences in the *SpPKC1 *promoter. To address this issue, we performed chromatin immunoprecipitation (ChIP) of chromatin prepared from late gastrula stage embryos using antibodies to either SpRunt-1 or SpCBFβ. Consistent with the expression data indicating that *CyIIIa *activation requires the heterodimer, sequences from the *CyIIIa *promoter are recovered by ChIP using both antibodies, as are sequences from *SpCyclinD *(Fig. 6B). In contrast, whereas sequences from the *SpPKC1 *promoter are recovered by ChIP using the SpRunt-1 antibody, they are not recovered by the SpCBFβ antibody (Fig. 6B). We conclude that in the gastrula stage embryo, SpCBFβ does not exist in a heterodimeric complex with SpRunt-1 within the context of the *SpPKC1 *promoter.

## Conclusion

Our data are consistent with the proposition that CBFβ is a facultative Runx partner that participates in the regulation of a subset of Runx target genes. It will be important to analyze the *SpPKC1 cis*-regulatory system to learn the contextual rules that facilitate CBFβ-independent Runx function, and to determine the biological rationale for Runx-mediated transcriptional regulation that does not involve CBFβ. One intriguing possibility is that genes that need to be rapidly responsive to physiological signals require Runx-DNA binding complexes that are less stable and/or have shorter half lives. Addressing this issue will require identification and comparison of the *cis*-regulatory systems from additional CFBβ-dependent and -independent Runx target genes.

## Methods

### Animals

Adult *Strongylocentrotus purpuratus *were obtained from Charles Hollahan (Santa Barbara Marine Biologicals, Santa Barbara, CA) or Pat Leahy (Corona del Mar, CA). Gamete collection and embryo culture were carried out as described previously [8].

### Microinjection and imaging of embryos

Embryo microinjections and imaging were carried out as previously described [8].

### Cloning of SpCBFβ

An alignment of all the known CBFβ protein sequences was used to design a degenerate PCR primer set. The following oligonucleotide primers were chosen to target conserved regions of the sequence alignment having amino acids with low codon degeneracy: N-terminal region MPRVVPDQ giving forward primer ATG CCI MGI GTI GTI CCI GAY CA, and central region NGVCV(LIR)W(RK)GW giving reverse primer CCA GGI ICK CCA IMD IAC RCA IAC ICC RTT (where M = A+C/Y = C+T/K = T+G/D = A+T+G/R = A+G). The SpCBFβ message was amplified from total RNA isolated from a 12 hour *S. purpuratus *embryo culture using 2-step PCR. Random primer was used in the cDNA synthesis step followed by the use of degenerate primers to direct PCR amplification of SpCBFβ from the random primed template. The single 350 bp PCR product, which was the size expected for a conserved sequence relative to the consensus, was desalted and cloned into plasmid vector pGEM T easy^® ^(Promega) by TA cloning. A set of 6 separate clones were fully sequenced and theoretical translation showed clear homology to consensus CBFβ. The nucleotide sequence of the partial SpCBFβ clone obtained by PCR with degenerate primers was in turn used to design specific primers for RACE. 5' RACE was performed starting with total RNA isolated from a 24 hour *S. purpuratus *embryo culture. Using GeneRacer™ (Invitrogen) following the manufacturer's protocol with the random primer option, 5' RACE cDNA was synthesized and used as a template for PCR with the included 5' RACE primer as a forward primer and a SpCBFβ-specific reverse primer. For 3' RACE, the phosphatase and ligation steps described in the GeneRacer™ protocol, designed to enrich for the 5' end, were eliminated. 3' RACE cDNA was synthesized with the oligo dT primer option and used as a template for PCR with an SpCBFβ specific forward primer, the included 3' RACE primer serving as the reverse primer. The final SpCBFβ sequence (Genbank accession number DQ205186) could not be extended further by RACE or database searches and is considered to be full-length.

### Recombinant protein production and electrophoretic mobility shift analysis (EMSA)

Full length SpRunt-1 and the Runt domain thereof were each subcloned from the original pSPORT1 plasmid into the bacterial expression vector pRSET A (Invitrogen). For subcloning, PCR primers were designed to insert the full length or partial coding sequence in frame relative to the his-tag encoded in the vector sequence. Likewise, the full coding region of SpCBFβ was subcloned into pRSET A downstream of the his-tag. For the SpRunt-1-G115R, base substitutions were made in the SpRunt-1 Runt domain/pRSET clone by the QuickChange^® ^II Site-Directed Mutagenesis method (Stratagene), using the following primers: Forward T ACG ATG GTG ACG ATC GCA GCA GGG AAC GAT GAA AAC T and Reverse T ACG ATG GTG ACG ATC GCA GCG CGC AAC GAT GAA AAC T. The based substitutions introduced a unique BssHll site for diagnostic purposes.

Each of the expression constructs was induced in a BL21-pLysS culture, and protein was isolated from the IPTG-induced culture using a Ni-NTA Spin Kit (Qiagen). EMSA was performed with fluorescently labeled oligonucleotides as previously described [8], using either the *CyIIIa *Runx site described previously [5] or the following Runx-site oligonucleotides as probe (from the *SpCyclinD *promoter region): 5'-/5Cy5/ATT ATT CTC TGA CCA CAA TTT TTG TTA GA-3' and 5'-TCT AAC AAA AAT TGT GGT CAG AGA ATA AT-3'.

### RNA blot analysis

RNA blot analysis was carried out on total RNA from staged embryos as described previously [6].

### Quantitative reverse transcription-coupled PCR (qRT-PCR)

For quantitation of relative transcript levels in control versus knockdown embryos, RT-PCR was performed as described previously [7], using RNA prepared from either 48 h (for SpPKC1, CyIIIa, and SpDri) or 36 h (for SpCyclinD) embryos. For quantitation of absolute transcript numbers, a SpCBFβ standard was prepared using the T7 mMessage Machine (Ambion) with the linearized clone as an in-vitro transcription template. After quantitation the SpCBFβ transcript was diluted in a 10 fold series ranging from 2.5 × 10^3 ^to 2.5 × 10^8 ^transcript copies and each dilution was adjusted to a final 5 μg amount by the addition of total RNA extracted from eggs (there is no endogenous SpCBFβ transcript detectable in eggs). Each of the diluted standards as well as 5 μg 24 and 40 hour total RNA samples were amplified by PCR using a 2-step procedure. First strand synthesis was performed using Superscript™ lll (Invitrogen) with the random primer option. PCR was then performed in a SmartCycler II (Cepheid) with SpCBFβ-specific primers and SYBR green detection. The final standard curve was used along with known values for total RNA yield per embryo and known number of embryos used for the sample RNA extraction to determine the transcript copy number per embryo.

### Antibody production and immunoblot analysis

The SpRunt-1 polyclonal antibody was described previously [5]. The SpCBFβ polyclonal antibody was similarly produced in rabbits by Cocalico Biologicals (Reamstown, PA), using purified recombinant SpCBFβ as antigen. The SLBP antibody was a gift of Dr. William Marzluff (University of North Carolina). Immunoblots were performed as described previously [7].

### Whole mount in situ hybridization (WMISH)

WMISH was performed by the methodology of Ransick and Davidson [25], as described in detail in [26].

### Morpholino antisense oligonucleotides

Morpholino antisense oligonucleotides (MASOs) were designed by and purchased from GeneTools, LLC (Corvallis, OR). The sequence of the SpCBFβ MASO is: CTACTCTGGGCATAGTTGACATCGG. The SpRunt-1 MASO m5 was described previously [7]. The standard non-specific control MASO from GeneTools was used as a negative control.

### Terminal transferase-mediated dUTP nick end labeling (TUNEL) assay

TUNEL assays on fixed embryos to image apoptosis were performed as previously described [8].

### mRNA synthesis

For rescue experiments, a mutation that changed Gly 115 to Arg was made in the SpRunt-1 full length rescue clone [7] by the QuickChange^® ^II Site-Directed Mutagenesis method (Stratagene), using the primers described above for the SpRunt-1 expression plasmid. The T7 mMessage Machine (Ambion) was used to synthesize full-length capped mRNA for microinjection.

### Chromatin immunoprecipitation (ChIP)

ChIP was carried out as previously described [8], using chromatin prepared from 48 h embryos and polyclonal IgG specific for SpRunt-1 and SpCBFβ. Non-immune or pre-immune IgG was used as a specificity control for anti-SpRunt-1 and anti-SpCBFβ antibodies, respectively. The following primers were used for PCR amplification ChIP samples: *SpPKC1*: GACCCCTGGCTTAATATGTTGATGTGTT (forward) and CCTTCATCTCAAACGAAGAATCCGACAT (reverse); *CyIIIa*: GTAGCACACGGAGAGATTGTGGGACAT (forward) and GGATCGGGGTTAGAGTTACATTTGGCTT (reverse); *SpCyclinD*: AGAAACGAATGTATCCGTGTGTTGTGAA (forward) and GCGAGACATAACTTCCTTGATCGTGCTA (reverse); and *SpCdk4*: CAGGAGCGTAGTCAATCCGCATCAA (forward) and CAGCCTGCAACTTCTGAGATGCTTTGT (reverse).

## Authors' contributions

AJR cloned the cDNA encoding SpCBFβ, made all the plasmid constructs, performed the RNA blot analysis, measured SpCBFβ transcript abundance using qRT-PCR, and assisted with ChIP. CD-S performed most of the microinjections, embryo labeling, confocal imaging and measurements of gene expression. AR performed the whole mount in situ hybridization. DER performed ChIP and EMSA experiments. JJM performed EMSA experiments and expressed the recombinant SpRunt-1 and SpCBFβ proteins. JAC directed the research, did some of the microinjections, acquired the DIC images, assisted with data analysis and drafted the manuscript and figures.
